# Utilisation of the National Early Warning Score (NEWS) and Assessment of Patient Outcomes Following Cardiac Surgery

**DOI:** 10.3390/jcm13226850

**Published:** 2024-11-14

**Authors:** Abiah Jacob, Azmi Qudsi, Niraj S. Kumar, Thomas Trevarthen, Wael I. Awad

**Affiliations:** 1 Department of Cardiac Surgery, Barts Heart Centre, St. Bartholomew’s Hospital, London EC1A 7BE, UK; abiah.jacob@nhs.net (A.J.);; 2 Department of Cardiovascular Sciences, University of Leicester, Leicester LE1 7RH, UK; 3 William Harvey Research Institute, Queen Mary University of London, London E1 4NS, UK; 4 University of South Wales, Cardiff CF24 2FN, UK

**Keywords:** national early warning score, cardiac surgery, predictive scores, outcomes

## Abstract

**Objectives:** The national early warning score (NEWS) was introduced to improve the detection of, and standardise the assessment of, the severity of acute illness in the National Health Service (NHS). We assessed whether the recommended threshold trigger score of 5 or more in a Critical Care Outreach Team (CCOT) review could accurately predict patients at risk of deterioration following cardiac surgery and patient outcomes. **Methods:** We investigated adult cardiac surgery patients between October 2019 and December 2021. NEWS 2 parameters triggering CCOT referrals and NEWS 2 parameters < 5 versus ≥5 were compared, and the resulting patient outcomes were evaluated. **Results:** Over this period, 3710 patients underwent surgery, of whom 162 (4.4%) initiated 193 calls to the CCOT. The mean number of NEWS 2 parameters on CCOT activation was 6.14 ± 2.43 (NEWS 0–16); 34 (20.98%) activations were from patients with NEWS 2 < 5. Low oxygen saturation (SpO_2_) (59.3%) and oxygen therapy (83.3%) were the most common physiological parameters raising the score. CCOT activations led to 38 transfers from the ward to the high-dependency unit (HDU) and 18 transfers to the intensive therapy unit (ITU). Cardiac arrest calls were initiated in 12 (7.40%) patients and two culminated in death. Fourteen (8.64%) had emergency resternotomy. The in-hospital mortality rate was 10.5% (17/162) in patients referred to CCOT versus 3.9% (139/3548) in patients who were not (*p* < 0.001). The in-hospital mortality in patients with NEWS 2 < 5 vs. NEWS ≥ 5 was 17.6% (6/34) versus 8.6% (11/128) (*p* = 0.126). **Conclusions:** There was no difference in in-hospital mortality in patients below or above a NEWS 2 of 5, but there was a significant difference in in-hospital mortality in patients reviewed by the CCOT (*p* < 0.001). Tailoring the threshold score specifically for the cardiac surgical cohort, in conjunction with clinician involvement, may improve outcomes.

## 1. Introduction

The national early warning score (NEWS) was launched in 2012 to detect and ensure a rapid clinical response to deteriorating patients in acute medical units across the NHS. The score was created to standardise the process of recording, scoring, and responding to changes in physiological parameters in acutely ill patients. It was established on the basis that early detection, timely action, and the capacity of the clinical response determine the clinical outcome in people with acute illness, and as such it has been widely implemented across the NHS and in other healthcare settings globally [1].

**Figure 1:**
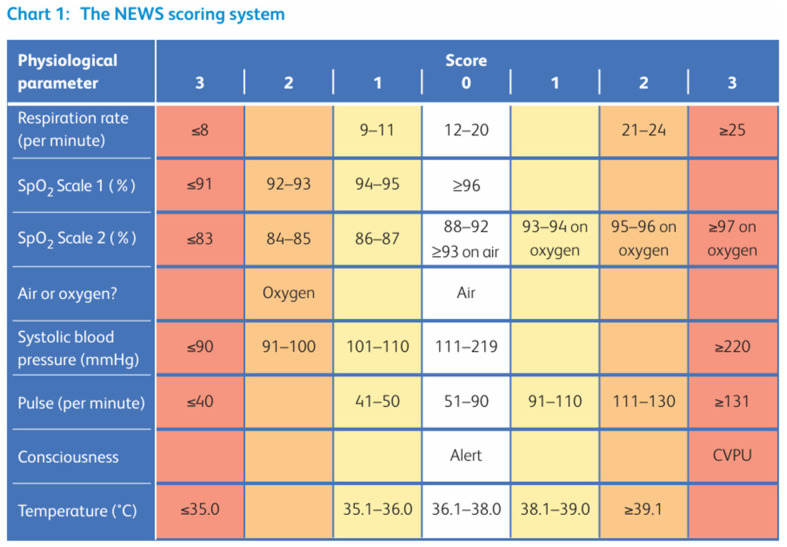
NEWS 2 score, adapted from the Royal College of Physicians [1]. SpO_2_ scale 2 is used in patients with Type 2 respiratory failure. CVPU—Confusion (new), Voice, Pain, Unresponsive. Reproduced from the Royal College of Physicians. National Early Warning Score (NEWS) 2: Standardising the assessment of acute-illness severity in the NHS. Updated report of a working party. London: RCP, 2017.

NEWS consists of a simple aggregate scoring system in which a score is allocated to physiological measurements which are already recorded in routine practice when patients are monitored in hospital. The score was updated in 2017 to NEWS 2 and the physiological parameters used were rearranged to align with the Resuscitation Council’s (UK) ABCDE sequence [2]. Six physiological parameters form the scoring system: respiration rate, oxygen saturation, systolic blood pressure, pulse rate, level of consciousness or new confusion, and temperature. Figure 1 details the full scoring system.

A score of 0, 1, 2, or 3 is allocated to each physiological parameter, with the magnitude of the score reflecting the variation from the norm, i.e., with 0 considered as the accepted norm range for these parameters and a score of 1, 2, or 3 being allocated in case of any deviation above or below this range. The higher the score assigned, the greater the deviation from the norm. There are two scales for oxygen saturation with a dedicated section (SpO_2_ Scale 2 with target SpO_2_ saturations between 88 and 92%) for patients with hypercapnic respiratory failure (HRF), usually observed in those with chronic obstructive pulmonary disease (COPD). Based on the Royal College of Physicians (RCP) guidelines, a NEWS of 5 or more is the key trigger threshold for urgent clinical review and action and also for considering serious sepsis in patients with a known or suspected infection [1].

The RCP states that NEWS should be used as an aid to clinical assessment rather than a substitute for competent clinical judgement and any concern about a patient’s clinical condition should prompt an urgent clinical evaluation, irrespective of the actual score [1].

NEWS has been validated in multiple settings ranging from ambulance services, emergency departments, intensive care units, acute care hospitals, and sepsis-related organ failure assessments to across wards [3,4,5,6,7]. However, the score does not wholly reflect whether the patient is improving or deteriorating, nor does it display the rate of improvement or deterioration over time. It only provides an immediate snapshot of the physiological parameters at any given point in time. Therefore, its utility in detecting a patient at risk of deterioration must only be taken into account based on the score at different time points.

Although the score was initially developed to serve as a tool for identifying acute medical deterioration, it has been evaluated in several surgical settings: general, colorectal, renal surgery, transplantation, urology, trauma, and orthopaedic surgery. In a study by Kovacs et al., the ability of NEWS to discriminate between cardiac arrest, death, and unanticipated ITU admission was evaluated in patients admitted to surgical specialties and the performance of the score was compared in patients admitted to medical specialties. The authors found that the score’s performance was equally good in non-elective surgical patients compared to non-elective medical patients [8]. Few studies have analysed NEWS 2 in the context of cardiac surgery, which is a specialty with a higher incidence of postoperative events compared to other surgical specialties.

With electronic records becoming increasingly ubiquitous across UK hospitals, the use of NEWS 2 as a bedside tool to assess and escalate acute illness has grown. While the score provides an immediate picture of deterioration, as observations are recorded and monitored at regular intervals, the predictive accuracy of NEWS 2 in cardiac surgery and its usage to call for an escalated clinical response requires further evaluation. Our study aims to assess the utility of NEWS 2 and the ability of the threshold trigger of 5 to adequately predict patients at risk of deterioration following cardiac surgery.

## 2. Materials and Methods

A retrospective study of all patients undergoing adult cardiac surgery at our centre with a Critical Care Outreach Team (CCOT) referral in the study period (between October 2019 and December 2021) was performed. Patients in the cardiac surgery wards postoperatively had their observations measured and recorded electronically using the Care Record Service (CRS) Millennium. Hospital databases were used to collect data on patient characteristics, operative details, and postoperative events leading to their CCOT referral, along with the NEWS 2 which led to their CCOT referral. NEWS 2 vital sign observations, including consciousness level, as well as the corresponding NEWS 2 scores were collated.

Patients who were referred for CCOT review were identified and their outcomes, such as postoperative stroke, postoperative sepsis, arrhythmias with haemodynamic compromise, escalation to the high-dependency unit or intensive therapy unit, resternotomy, and in-hospital mortality, were investigated. At our centre, a CCOT referral is made for patients in whom clinical deterioration is witnessed as determined by the ward team of doctors, or in those with a NEWS 2 of ≥5. The outcomes of patients after their CCOT review were assessed, and patients with NEWS 2 below the threshold trigger of 5 versus those with a score of ≥5 and above were compared.

### Data Analysis

We conducted an analysis comparing patients referred to the CCOT, together with their NEWS 2 (NEWS 2 < 5 and NEWS 2 > 5) at the time of referral, to those not referred to the CCOT. Continuous variables were compared with an unpaired 2-sample *t*-test, while binary outcomes were assessed using a chi-square test. We summarised the demographic characteristics of the patients in our sample with descriptive statistics, including the mean ± one standard deviation (SD). A logistic regression model was used to assess whether a cutoff of the NEWS 2, i.e., 5, could predict in-hospital mortality by comparing patients with a CCOT referral above and below a NEWS 2 of 5. A *p*-value of 0.05 was considered statistically significant. The data were organised and coded using Microsoft Excel and analysed using SPSS version 16.

## 3. Results

A total of 3710 patients underwent cardiac surgery during the study period, of whom 162 (4.37%) were referred to the CCOT for review. In these patients, the mean age was 65.59 ± 12.12 years, 114 (70.37%) were male, and the mean EuroSCORE II was 5.87 ± 7.20.

The patient characteristics of those referred to the CCOT versus those who were not referred are summarised in Table 1.

Patients escalated for CCOT review (*n* = 162) were more likely to be haemodialysis-dependent preoperatively (5.6% vs. 1.3%) and have extracardiac arteriopathy (11.1% vs. 5.6%) and a higher EuroSCORE II score (5.87 ± 7.20 vs. 3.67 ± 6.37). In addition, patients referred for CCOT review had a higher in-hospital mortality (10.5% vs. 3.9%, *p* < 0.001) than those patients who were not referred to the CCOT.

The operative details of patients who were reviewed by the CCOT versus those who were not are summarised in Table 2.

The majority of patients reviewed by the CCOT had undergone elective surgery (46.6%) and isolated coronary artery bypass grafts (CABG) (37.04%).

**Figure 2:**
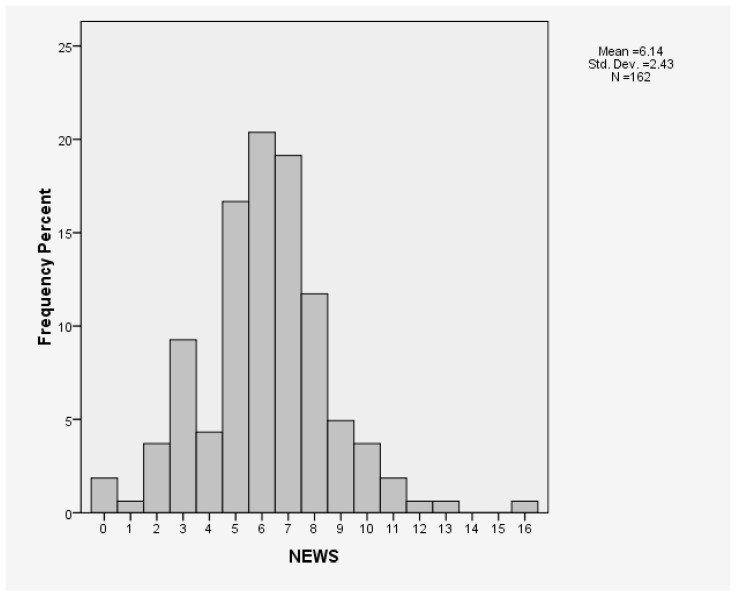
Distribution of NEWS at activation in patients referred to CCOT.

Among the patients who underwent a CCOT review, their mean NEWS 2 score upon CCOT activation was 6.12 ± 2.43 (NEWS 2 between 0 and 16), with 34 (21.0%) activations from patients with a NEWS 2 < 5 (Table 3). Figure 2 shows the distribution of NEWS 2 amongst patients for whom the CCOT was activated.

The parameters responsible for raised NEWS 2 scores are summarised in Table 3.

The most common NEWS 2 parameters contributing to activations were the need for oxygen therapy via a nasal cannula or face mask in 135 (83.3%), low SpO_2_ in 96 (59.3%), and low systolic blood pressure in 90 (55.6%).

CCOT referrals led to 38 (23.5%) transfers from the ward to the HDU and 18 (11.1%) transfers from the ward to the ITU, while the remaining 106 (65.4%) patients remained on the ward. Nine of the thirty-eight patients (23.6%) that were transferred to the HDU were later escalated from the HDU to the ITU for further management. A total of 12 of the 162 (7.4%) patients triggered a cardiac arrest call, and two arrests led to death on the ward; 14 of 162 (8.6%) required an emergency resternotomy. The mean length of hospital stay in these patients was 22.84 ± 25.18 days and 17 of the 162 (10.5%) patients died in hospital.

In total, 27 (16.7%) patients were transferred to the ITU following their CCOT review; 18 (66.7%) from the ward and 9 (33.3%) after being transferred to the HDU. Six patients (22.2%) who were transferred to the ITU had a NEWS 2 < 5, while eight patients who were transferred to the HDU (21.1%) had a NEWS 2 < 5. The most common reasons for escalation to the ITU were respiratory failure, seen in 9/27 (33.3%) patients, and persistent hypotension, seen in 6/27 patients (22.2%). The most common reasons for escalation to the HDU were for the management of respiratory distress, seen in 12/38 (31.6%) patients, and arrhythmias with haemodynamic compromise, seen in 10/38 (26.3%) patients. The results are summarised in Table 4.

New filtration was initiated in 9/34 (26.5%) versus 18/128 patients (14.1%, *p* = 0.136) postoperatively (these patients included haemodialysis-dependent patients with chronic kidney disease (9, 5.6%) and patients who developed acute kidney injury postoperatively (153, 94.4%)). Patients with a NEWS 2 ≥ 5 (41, 32.0%) had a higher rate of postoperative sepsis when compared with those with a NEWS 2 < 5 (5, 14.7%) (*p* = 0.046).

The evaluation of outcomes between patients who had NEWS 2 above and below the trigger threshold of 5 via logistic regression revealed that the NEWS 2 score alone is not a significant predictor of in-hospital death (OR = 1.010, 95% CI [0.990, 1.031], *p* = 0.308).

## 4. Discussion

This retrospective study evaluates the effectiveness of NEWS 2 in detecting patients at risk of deterioration following cardiac surgery. We have shown that NEWS 2 is a reliable tool in recognising postoperative sepsis, with patients who had a NEWS 2 of 5 or more having a higher rate of sepsis compared to those with a NEWS 2 < 5 (*p* = 0.046). This is consistent with findings by others who have studied and validated this score in medical and surgical hospital wards, the emergency department, and in pre-hospital settings, where the score was found to facilitate the early recognition and treatment of deteriorating and septic patients and was also a reliable predictor of mortality [9,10,11]. Durr et al. compared the performances of NEWS and the quick Sepsis-Related Organ Failure score (qSOFA) for the detection of sepsis in patients admitted to the emergency department and found that a NEWS > 5 has a better sensitivity (86% versus 34%), accuracy (72% versus 60%), and negative predictive value (77% versus 54%) than the qSOFA for the early detection of sepsis in the emergency department, although the qSOFA had a better positive predictive value (80% versus 69%). NEWS was also superior to the qSOFA in predicting ICU admission and 28-day mortality, 82% versus 33% and 88% versus 37%, respectively [12].

In our cohort, although in-hospital mortality was understandably higher amongst those patients who underwent CCOT review and had a NEWS 2 ≥ 5 vs. NEWS 2 < 5, a logistic regression analysis showed that NEWS 2 alone was not a significant predictor of in-hospital mortality. This is similar to the findings from Dundar et al., who found that NEWS is not an accurate predictor of in-hospital mortality in geriatric ED patients who are critically ill [13]. However, the NEWS risk classification was found to be a reliable predictor of in-hospital mortality in multiple other settings (pre-hospital, emergency department, and ward settings) by identifying deteriorating patients. In these settings, an elevated NEWS was associated with a higher incidence of adverse outcomes with an increased rate of mortality. Additionally, NEWS helped to facilitate the screening, earlier recognition, and treatment of sepsis in deteriorating patients [9,10,11].

Despite an aggregate score of 5 or more being the threshold score for an urgent clinical review, 34 CCOT activations were noted from patients with a NEWS 2 < 5. This corroborates the statements by Williams in their review of NEWS/NEWS 2, who emphasised that even if the score is low, care must be escalated if a healthcare professional or carer is concerned about a patient. Although NEWS is a powerful tool for assessment, it is not a substitute for sound clinical judgement or intuition which may indicate that a patient is deteriorating. Williams also discussed the contribution of vital sign physiology towards NEWS and the basis for the escalation of care, stating that acutely ill patients may not always have a marked disturbance in their vital signs in proportion to their need for an urgent escalation of care [14]. This is in keeping with 54.8% of our patients who were escalated for a CCOT review despite a NEWS 2 < 5 and no marked changes in their vitals and had escalation of their care to the critical care unit. NEWS is a cross-sectional picture of vital signs and is not an indication of patient improvement or deterioration. After cardiac surgery, patients are at a higher risk of postoperative complications such as postoperative atrial fibrillation (POAF), which has a 20–40% incidence after coronary artery bypass surgery and is associated with a higher risk of cerebrovascular accidents and a higher risk of cardiovascular and all-cause mortality [15].

Our cohort referred to the CCOT also had a higher cardiac operative risk (EUROSCORE II 5.87 vs. 3.67, *p* < 0.001) compared to patients not referred to the CCOT. The EuroSCORE was also found to be a good predictor of major postoperative morbidity in cardiac surgery with regard to respiratory and dialysis-dependent renal failure [16,17]. Hence, patients who undergo cardiac surgery are at a higher risk of postoperative complications such as acute kidney injury requiring dialysis, stroke, and in-hospital mortality, indicating that a high risk of clinical suspicion and a low threshold for the escalation of care is required in this patient population. As such, our study suggests that combining risk stratification scores such as the EUROSCORE II with early warning scores such as the NEWS 2 may lead to improved outcomes via the frequent monitoring of patients who are at a higher risk of developing postoperative complications (i.e., patients with a high preoperative EUROSCORE II).

Patients may also benefit from adjustments in the thresholds of NEWS 2 for an escalation of care based on their clinical status, which may involve altering the individual alert levels in these patients, as suggested by Subbe et al. in their recommendations for maximising the impact of NEWS [18].

Another area where individual alert alterations would be beneficial in our patients is oxygen therapy and saturation, which were the most common physiological parameters raising the score and contributing to CCOT activations in our patients. Since most of our postoperative surgical patients routinely receive supplemental oxygen therapy, it could induce a higher score that leads to a falsely elevated NEWS 2, triggering an unwarranted CCOT review.

In a retrospective cohort study, Alhmoud et al. investigated the performance of the digital NEWS 2 in predicting critical events in a cardiac specialist setting with regard to death, intensive care unit (ICU) admission, cardiac arrest, and medical emergencies. They found that the performance of NEWS 2 in patients with cardiovascular disease is suboptimal in predicting early deterioration, and an adjustment of the score using variables which strongly correlate with critical cardiovascular outcomes can improve early scoring models and help strengthen the prognostic performance of the tool. They authors stated that clinicians’ involvement in models’ development and validation would help to produce a higher accuracy warning score in conjunction with clinical expertise [19].

One of the main drawbacks of NEWS is that it requires trained professionals to calculate the score; it is time-consuming and prone to calculation error, and a mere recording of the scores, despite its predictive ability, will not lead to improved outcomes unless timely intervention is undertaken [14,20]. Williams, in their review, also stated that one of the factors that would help to improve the outcomes of patients with acute illness/deterioration is the competency of the responder (s) [14]. Thus, it is essential to have well-trained staff with adequate experience that can recognise acute patient deterioration and escalate care in a timely manner, especially in cardiac surgical cohorts.

Although NEWS 2 reliably predicted postoperative sepsis in our patient cohort, where patients with a NEWS 2 ≥ 5 (*n* = 41, 32.03%) had a higher rate of sepsis compared with those with a NEWS 2 < 5 (*n* = 5, 14.71%), it is suboptimal in other areas within the cardiac surgery setting. The score would benefit from tailored modifications specific to this specialty in conjunction with the five ‘R’s of Rapid Response Systems. These include the elements of a monitoring system aimed at preventing patient harm: the recording of physiological observations, recognising the degree of abnormality, reporting to a skilled healthcare professional, responding with the appropriate treatment, and repetition of the previous four steps as a feedback loop to identify failures in clinical improvement [21].

Our study should be considered in the context of certain limitations. We are presenting a retrospective analysis of a cohort from our centre, which was composed of a relatively small sample of patients. Thus, our results have limited power and are subject to confounding. Further prospective multicentre studies should be conducted to validate our findings in the cardiac surgery population.

## 5. Conclusions

We have shown that there is no significant difference in patient mortality above or below the threshold score of 5. However, the score aided in identifying patients who developed postoperative sepsis. Although the NEWS 2 provides a common language for the assessment of clinical severity and can be used as a tool to trigger clinical intervention, the threshold score of 5 as a marker for clinical escalation is inadequate in cardiac surgery patients since this patient cohort is at a higher risk for postoperative complications. The benefits of NEWS 2 may be enhanced if it is used as an adjunct to other risk assessment scores, such as the EUROScore 2, along with a lower escalation threshold and frequent monitoring, as determined by the surgical team. Future research should be directed towards creating a specialty-specific score for the cardiac surgical cohort with clinician involvement.

## Figures and Tables

**Table 1:** Summary of patient characteristics.

Variable	Patients Reviewed by CCOT *n* = 162	Patients Not Reviewed by CCOT *n* = 3548	*p* Value
Age (years) (mean ± SD)	65.59 ± 12.12	62.39 ± 12.433	0.001 **
Male	114 (70.4%)	2667 (75.2%)	0.168
LVEF	52.42 ± 10.301	51.86 ± 9.458	0.507
CRF needing dialysis pre-op	9 (5.6%)	46 (1.3%)	<0.001 **
COPD	9 (5.6%)	348 (9.8%)	0.076
DM	32 (19.9%)	1033 (29.1%)	0.011 **
Extracardiac Arteriopathy	11.1% (*n* = 18)	5.6% (*n* = 199)	<0.001 **
Hypertension	77.2% (*n* = 125)	75.2% (*n* = 2667)	0.291
EUROSCORE II	5.87 ± 7.20	3.67 ± 6.37	<0.001 **
In-hospital Mortality	10.49% (*n* = 17)	3.92% (*n* = 139)	<0.001 **

** Statistically significant finding (*p* < 0.05). LVEF = left ventricular ejection fraction (Hyperdynamic LVEF: >70%; Normal LVEF: 50–70%; Mild dysfunction LVEF: 40–49%; Moderate LVEF 30–39%; Severe LVEF < 30%); CRF = chronic renal failure; COPD = chronic obstructive pulmonary disease; DM = diabetes mellitus.

**Table 2:** Summary of operative details of all patients.

Operative Details	Patients Receiving CCOT Review, *n*/162 (%)	Patients Without CCOT Review, *n*/3548 (%)	*p* Value
Elective	75 (46.6%)	1889 (53.2%)	0.083
Urgent	60 (37.0%)	1403 (39.5%)	0.523
Emergency	27 (16.7%)	231 (6.5%)	<0.001 **
Salvage	0 (0%)	25 (0.7%)	0.284
Redo	14 (8.6%)	201 (5.7%)	0.108
Isolated CABG	60 (37.04%)	1751 (49.36%)	0.002 **
Isolated Valve	31 (19.14%)	1000 (28.18%)	0.012 **
CABG + Valve	18 (11.11%)	314 (8.85%)	0.324
Other Procedures (aortic, vascular surgery, septal myectomy, congenital)	53 (32.71%)	483 (13.61%)	<0.001 **

** Statistically significant finding (*p* < 0.05). CABG = coronary artery bypass graft; Valve = operations involving any of the heart valves; other procedures = septal and aortic/vascular procedures.

**Table 3:** NEWS 2 scores of patients by individual physiological parameter.

NEWS Parameter	% of Patients with Raised NEWS in This Parameter *n* (%)
Respiratory rate	67 (41.4%)
SpO_2_	96 (59.3%)
Oxygen therapy	135 (83.3%)
Systolic blood pressure	90 (55.6%)
Pulse rate	87 (53.7%)
Alertness (CAVPU)	10 (6.2%)
Temperature	40 (24.7%)

SD = standard deviation; SpO_2_ = oxygen saturation.

**Table 4:** Assessing the threshold of 5 for NEWS 2 among patients reviewed by the CCOT.

Variable	NEWS 0–4 (*n* = 34)	NEWS ≥ 5 (*n* = 128)	*p* Value
In-Hospital Death	6 (17.65%)	11 (8.59%)	0.126
Postoperative Stroke	3 (8.82%)	8 (6.25%)	0.596
Preoperative Dialysis	2 (5.88%)	7 (5.47%)	0.136
Escalated to HDU/ITU	14 (41.17%)	43 (33.59%)	0.456
Developed Postoperative Sepsis	5 (14.71%)	41 (32.03%)	0.046 **
Resternotomy	4 (11.76%)	10 (7.81%)	0.111

** Statistically significant finding (*p* < 0.05).

## Data Availability

The original contributions presented in this study are included in this article. Further inquiries can be directed to the corresponding author.
